# In vivo antileishmanial activity and chemical profile of polar extract from *Selaginella sellowii*

**DOI:** 10.1590/0074-02760150307

**Published:** 2016-03

**Authors:** Dayane Priscilla de Souza Queiroz, Carlos Alexandre Carollo, Mônica Cristina Toffoli Kadri, Yasmin Silva Rizk, Vanessa Carneiro Pereira de Araujo, Paulo Eduardo de Oliveira Monteiro, Patrik Oening Rodrigues, Elisa Teruya Oshiro, Maria de Fátima Cepa Matos, Carla Cardozo Pinto de Arruda

**Affiliations:** 1 Universidade Federal de Mato Grosso do Sul, Centro de Ciências Biológicas e da Saúde, Laboratório de Parasitologia Humana, Campo Grande, MS, Brasil; 2 Universidade Federal de Mato Grosso do Sul, Centro de Ciências Biológicas e da Saúde, Laboratório de Produtos Naturais e Espectrometria de Massas, Campo Grande, MS, Brasil; 3 Universidade Federal de Mato Grosso do Sul, Centro de Ciências Biológicas e da Saúde, Laboratório de Biofisiofarmacologia, Campo Grande, MS, Brasil; 4 Universidade Federal de Mato Grosso do Sul, Centro de Ciências Biológicas e da Saúde, Laboratório de Tecnologia Farmacêutica, Campo Grande, MS, Brasil; 5 Universidade Federal de Mato Grosso do Sul, Centro de Ciências Biológicas e da Saúde, Laboratório de Biologia Molecular e Culturas Celulares, Campo Grande, MS, Brasil

**Keywords:** antileishmanial activity, plant extracts, natural products, experimental leishmaniasis

## Abstract

The polar hydroethanolic extract from *Selaginella sellowii*(SSPHE) has been previously proven active on intracellular amastigotes (in vitro test) and now was tested on hamsters infected with *Leishmania (Leishmania) amazonensis* (in vivo test). SSPHE suppressed a 100% of the parasite load in the infection site and draining lymph nodes at an intralesional dose of 50 mg/kg/day × 5, which was similar to the results observed in hamsters treated with *N*-methylglucamine antimonate (Sb) (28 mg/Kg/day × 5). When orally administered, SSPHE (50 mg/kg/day × 20) suppressed 99.2% of the parasite load in infected footpads, while Sb suppressed 98.5%. SSPHE also enhanced the release of nitric oxide through the intralesional route in comparison to Sb. The chemical fingerprint of SSPHE by high-performance liquid chromatography with diode-array detection and tandem mass spectrometry showed the presence of biflavonoids and high molecular weight phenylpropanoid glycosides. These compounds may have a synergistic action in vivo. Histopathological study revealed that the intralesional treatment with SSPHE induced an intense inflammatory infiltrate, composed mainly of mononuclear cells. The present findings reinforce the potential of this natural product as a source of future drug candidates for American cutaneous leishmaniasis.

American cutaneous leishmaniasis (ACL) is an infectious, noncontagious disease caused by different species of protozoa of the genus *Leishmania* Ross, 1903, that affects the skin, cartilage, and mucous membranes of the upper respiratory tract (Reithinger et al. 2007). Drugs used in the treatment of leishmaniasis have a number of drawbacks, such as high degrees of toxicity, the development of resistance on the part of the parasite, and high costs (Santos et al. 2008). Pentavalent antimonials are the first choice for treatment while other drugs, such as pentamidine, amphotericin B, and paromomycin are used as a second option in resistant cases, despite the considerable degree of toxicity to the host (Mitropoulos et al. 2010).

A number of plant-derived extracts have been tested in experimental leishmaniasis, looking for the better effects and less toxicity showed by these natural products (Fournet et al. 1996, Pontin et al. 2008, Ezatpour et al. 2015). Different secondary metabolites with considerable structural variety have demonstrated antileishmanial activity while offering a low degree of toxicity and allowing other forms of administration, such as derivatives of hydroquinones, naphthoquinones, terpenoids, flavonoids, alkaloids, and lignans (Fournet & Muñoz 2002). Recently, the hydroethanolic extract from*Selaginella sellowii* was proven active on *Leishmania (Leishmania) amazonensis* intracellular amastigotes (Rizk et al. 2014). This noncytotoxic extract contained amentoflavone and robustaflavone, two compounds of the main bioactive class in*Selaginella* genus, the biflavonoids (Lin et al. 1994, Silva et al. 1995, Sun et al. 1997, Aguilar et al. 2008, 20, 2013, Lee et al. 2008).

The aim of the present study was to investigate the in vivo antileishmanial activity of the hydroethanolic extract from *S. sellowii* in hamsters, a susceptible model for experimental cutaneous leishmaniasis, where it was administered by intralesional and oral route.

## MATERIALS AND METHODS

*Animals* - Male golden hamsters (*Mesocricetus auratus*) aged 30-40 days were used as the experimental model of infection. The animals were obtained from the central animal facility of the Centre for Biological and Health Sciences (CCBS) of the Federal University of Mato Grosso do Sul (UFMS), state of Mato Grosso do Sul (MS), Brazil in good health and free of infections or parasites common to rodents, maintained in individually ventilated cages equipped with mini-isolators, fed a balanced feed (Nuvilab CR-1; Nuvital, Brazil) with free access to water. This study received approval from the local Animal Experimentation Ethical Committee (UFMS) under protocol 402/2012.

*Plant material* - Plant specimens of *S. sellowii*Hieron. 1990 (Selaginellales: Selaginellaceae) were collected in MS, in June 2009. Voucher material was deposited in the CGMS Herbarium/UFMS under registration 27218 (Genetic Heritage Management Council/Brazilian Ministry of the Environment license 010273/2013-1), after identification by Dr Arnildo Pott (Botany Laboratory, CCBS/UFMS). Crude extract was obtained from the whole dried pulverised plant. Plant material (66 g) was extracted in a pressurised liquid extractor (ASE-150; Dionex, USA), first with dichloromethane to remove apolar compounds, followed by a mixture of ethyl acetate:methanol (8:2) and finally ethanol:water (7:3), obtaining the hydroethanolic extract - polar hydroethanolic extract from *S. sellowii*(SSPHE) with yield of 8.9% (w/w) (Rizk et al. 2014). SSPHE was endotoxin free.

*Fingerprint of SSPHE by high-performance liquid chromatography with diode-array detection and tandem mass spectrometry (HPLC-DAD-MS/MS)* - The SSPHE was solubilised in methanol:water 1:1 (2 mg/mL) and a 2 µL sample was injected in an Ultra Fast Liquid Chromatograph Shimadzu LC-20AD coupled with a DAD and ESI-qTOF microTOF-Q III (BrukerDaltonics, USA) detectors coupled in-line. The DAD was monitoring between 240-800 and mass spectrometer operates in negative mode (120-1200 Da and collision energy 45-65 V). The stationary and mobile phases were a C-18 column (2.6 μ, 150 x 2.2 mm) (Kinetex, USA) protected by a pre-column with the same material, a gradient elution program using water (phase A) and acetonitrile (phase B), both with 1% of acetic acid: 0-2 min, 3% of B; 2-25 min, 3-25% of B; 25-40 min, 25-80% of B, followed by washing and reconditioning of the column (8 min). Flow rate: 0.3 mL/min. The compounds amentoflavone and robustaflavone were identified by comparison with standards (Rizk et al. 2014). Other compounds were putatively identified, based on their molecular mass, fragmentation, and ultraviolet (UV) spectrum.

*Parasites* - A standard strain of *L. (L.) amazonensis* (IFLA/BR/1967/PH8) was used for the establishment of infection. Promastigote forms were cultured at 25ºC in Schneider’s Insect Medium (Sigma, USA) supplemented with 20% foetal calf serum (FCS) (Cultilab, Brazil) and 140 µg/mL gentamicin (Sigma). The parasites were maintained in vivo through serial passages in hamsters (*M. auratus*).

*Infection and treatment of infected animals* - Ninety animals were infected subcutaneously in the left hind footpad with 1 x 10^6^ *L. amazonensis* promastigotes. Treatment began 28 days post-infection when the infection was well established. The animals were divided into six groups according to the route of administration and type of treatment. The groups treated through the intralesional route received five injections of SSPHE [50 mg/kg in 0.05 mL phosphate-buffered saline (PBS)/Tween 80 10%], PBS/Tween 80 10% or*N-*methylglucamine antimonate (Sb) (Glucantime^®^; Sanofi-Aventis, Brazil) (28 mg/kg), respectively, in the infection site with a four-day interval between administrations. The groups submitted to oral administration received 0.2 mL of SSPHE (50 mg/kg/day in PBS/Tween 80 10%), Sb (28 mg/kg/ day), or PBS/Tween 10% by gavage, daily, for 20 days.

*Evaluation of effects* - The kinetics of the cutaneous lesion was evaluated weekly after infection until one week after the end of treatment. Footpad thickness was measured using a caliper with an accuracy of 0.01 mm (Worker, Brazil) and was expressed as the difference between the infected footpad and the mean of five noninfected footpads.

Parasite load was evaluated at the inoculation site and popliteal draining lymph nodes one week after the end of treatment. The organs were removed, weighed, and homogenised in 1 mL of Schneider’s Insect Medium (Sigma) supplemented with 20% FCS (Sigma) and 140 µg/mL gentamicin (Sigma). The limiting dilution assay was performed in duplicate, as previously described (Titus et al. 1985). The parasite load was calculated using the geometric mean reciprocal of positive titres obtained for the homogenate of each organ divided by the respective weight and the number of parasites per nanogram of tissue was then calculated. The parasite suppression index (SI) was calculated using the following formula:


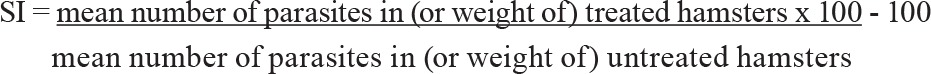


*Nitric oxide (NO) evaluation* - Cells obtained from the peritoneum of control and treated animals were collected, quantified, and resuspended in RPMI-1640 medium (Sigma) supplemented with 10% FCS (Gibco, USA) and 140 µg/mL gentamicin (Sigma) at a concentration of 1 x 10^5^ mL^-1^. Cells were incubated for 48 h at 37ºC in a humid atmosphere containing 5% CO_2_. Afterwards, 100 µL of the supernatants were collected and incubated with an equal volume of Griess reagent (1% sulfanilamide/0.1% naphthalene diamine dihydrochloride/2.5% H_3_PO_4_) for 10 min at room temperature for the quantification of the accumulation of nitrite (Ding et al. 1988). Absorbance was determined at 540 nm. The conversion to µM of NO_2_^-^ was obtained by comparing the samples to a standard curve obtained with known concentrations (1-10 µM) of sodium nitrite diluted in RPMI medium.

*Histopathological study* - Infected and treated footpads were removed and fixed in 10% buffered formalin for subsequent embedment in paraffin. Sections (5 µm) were performed on a microtome (Zeiss Hyrax M25) and stained with haematoxylin-eosin. Photomicrographs were taken on an image capturing microscope (Leica DM5500B); the nature of the inflammatory infiltrate and the presence of parasites were analysed.

*Statistical analysis* - Footpad thickness and NO production were expressed as the mean ± standard deviation (SD) of 15 and five animals per group, respectively, and the data were analysed using the Student’s *t*test. Organ weights were expressed as the mean ± SD of five animals per group and the data were analysed using ANOVA, followed by Tukey post-test. Differences were considered significant at p < 0.05 (represented by an asterisk).

## RESULTS

**Fig. 1A:**
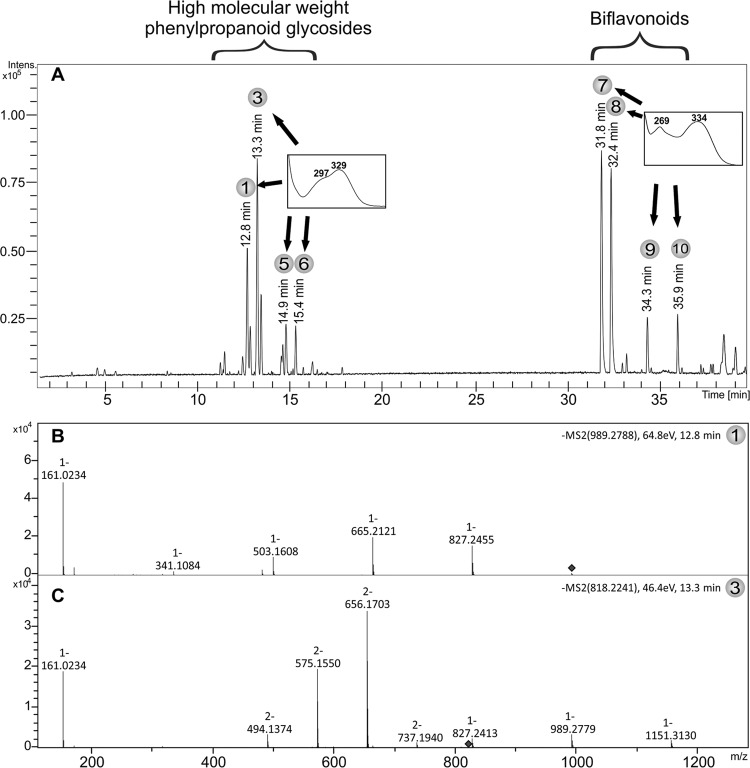
: high-performance liquid chromatography-mass spectrometry chromatographic profile and ultraviolet absorption spectra of the polar hydroethanolic extract from *Selaginella sellowii*; B: fragmentation of the peak 12.8 min *m/z* 989.2788 with an energy collision of 64.8 eV; C: fragmentation of the peak 13.3 min*m/z* 818.2241 with an energy collision of 46.4 eV. 1: dicaffeoyl-O-tetra-hexoside; 3: tetra-caffeoyl-O-hexa-hexoside; 5: tetra-caffeoyl-O-hexa-hexoside; 6: tetra-caffeoyl-O-penta-hexoside; 7: amentoflavone; 8: robustaflavone; 9: hinokiflavone; 10: OMe-hinokiflavone.

*Fingerprint of SSPHE* - HPLC-DAD-MS/MS analysis of SSPHE showed the presence of two classes of compounds: biflavonoids and caffeoyl-hexoside derivatives of high molecular weight (Fig. 1A). The main biflavonoids, amentoflavone, and robustaflavone were identified in a previous work (Rizk et al. 2014); now two new biflavonoids are observed (Table I). The peak at 34.3 min with an *m/z*of537.0821 [M-H]^-^ is compatible with the formula C_30_H_18_O_10_ (537.0827, error 1.2 ppm). The fragmentation of *m/z* 537 generates the ions at*m/z* 284 (C_15_H_8_O_6_) and*m/z* 269 (C_15_H_9_O_5_). This peak was putatively identified as hinokiflavone based on their fragments and UV spectrum and the peak at 35.9 min *m/z* 551.0977 (compatible with the formula C_31_H_20_O_10_ - 551.0984, error 1.3 ppm) showed a similar UV spectrum and fragments of 34.3 min and was putatively identified as OMe-hinokiflavone.

**TABLE I:** Retention times (Rt), maximal absorption wavelength (UV/VIS), formula, and molecular weight (m/z) of compounds of the polar hydroethanolic extract from *Selaginella sellowii*(SSPHE)

Rt	UV/VIS	Molecular formula	[M-H]^-^(m/z)	MS/MS (m/z)	Compound
12.8	297/329	C_42_H_54_O_27_	989.2789	827 (C_33_H_47_O_24_), 665 (C_23_H_41_O_21_), 503 (C_18_H_31_O_16_), 341 (C_12_H_21_O_11_), 161 (C_9_H_5_O_3_)	putative dicaffeoyl-*O*-tetra-hexoside
13.0	297/329	C_42_H_54_O_27_	989.2785	827 (C_33_H_47_O_24_), 665 (C_23_H_41_O_21_), 503 (C_18_H_31_O_16_), 341 (C_12_H_21_O_11_), 161 (C_9_H_5_O_3_)	putative dicaffeoyl-*O*-tetra-hexoside
13.3	297/324	C_72_H_86_O_43_	818.2243^*a*^	1151 (C_51_H_59_O_30_), 989 (C_42_H_53_O_27_), 827 (C_33_H_47_O_24_), 161 (C_9_H_5_O_3_)	putative tetra-caffeoyl-*O*-hexa-hexoside
13.5	297/324	C_57_H_70_O_35_	656.1809^*a*^	989 (C_42_H_53_O_27_), 827 (C_33_H_47_O_24_), 665 (C_23_H_41_O_21_), 161 (C_9_H_5_O_3_)	putative tri-caffeoyl-*O*-penta-hexoside
14.9	297/324	C_72_H_86_O_43_	818.2233^*a*^	1151 (C_51_H_59_O_30_), 989 (C_42_H_53_O_27_), 827 (C_33_H_47_O_24_), 161 (C_9_H_5_O_3_)	putative tetra-caffeoyl-*O*-hexa-hexoside
15.4	297/324	C_66_H_76_O_38_	737.1964^*a*^	1151 (C_51_H_59_O_30_), 989 (C_42_H_53_O_27_), 827 (C_33_H_47_O_24_), 665 (C_23_H_41_O_21_), 161 (C_9_H_5_O_3_)	putative tetra-caffeoyl-*O*-penta-hexoside
31.8	269/334	C_30_H_18_O_10_	537.0828	375 (C_21_H_11_O_7_), 331 (C_20_H_11_O_5_)	amentoflavone
32.4	269/334	C_30_H_18_O_10_	537.0819	331 (C_20_H_11_O_5_), 309 (C_17_H_9_O_6_)	robustaflavone
34.3	269/334	C_30_H_18_O_10_	537.0821	284 (C_15_H_8_O_6_), 269 (C_15_H_9_O_5_)	putative hinokiflavone
35.9	269/334	C_31_H_20_O_10_	551.0977	283 (C_15_H_7_O_6_), 255 (C_14_H_7_O_5_)	putative OMe-hinokiflavone

*a*: [M-H]^-2^; MS/MS: tandem mass spectrometry .

Polar compounds between 11-17 min were also observed in the chromatogram. These compounds showed an UV spectrum characteristic of the caffeoyl/feruloyl group (Grassi-Zampieron et al. 2010) and a molecular weight range of 990-1638 Da (Table I). The fragmentation patterns of these peaks are similar to the sequential losses of caffeoyl acids and hexose moieties (Fig. 1B,C). The compounds were putatively determined as di, tri, or tetracaffeoyl acids with tetra, penta, or hexahexosides, based on the molecular formula and fragmentation; however, the groups’ position could not be determined. Molecular weight, formula, and fragmentation of these compounds are shown in the Table I.

**Fig. 2:**
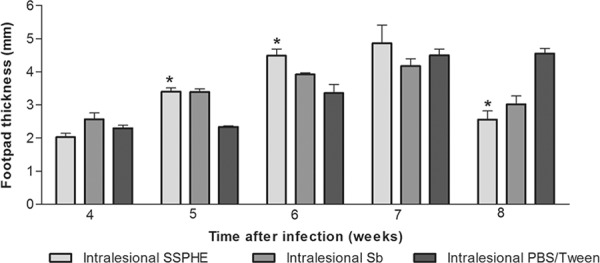
: kinetics of cutaneous lesion induced by *Leishmania amazonensis* after treatment with polar hydroethanolic extract from *Selaginella sellowii* (SSPHE) administered*via* intralesional injection (5 injections of 50 mg/kg with intervals of 4 days). Controls received*N*-methylglucamine antimonate (Sb) or phosphate-buffered saline (PBS)/Tween by the same route. Hamsters were infected in the left hind footpad with *L. amazonensis* promastigotes and treatment started four weeks after infection, ending seven weeks after infection. The data represent the mean ± standard deviation of 15 animals per group. Asterisk means p < 0.05 for SSPHE-treated*vs.* control animals (PBS/Tween). Student’s*t* test.

*Effect of SSPHE throughout progression of cutaneous lesion* -*L. amazonensis* promastigotes induced a progressive increase in thickness of the infected footpad in most hamsters. Intralesional treatment with SSPHE resulted in progressively greater thickness towards the end of treatment in comparison to the group that received only PBS/Tween by the same administration route (Fig. 2). However, thickness of the footpads treated with SSPHE was significantly reduced one week after the end of the treatment in comparison to untreated footpads. Sb administered by the same route also induced a gradual increase in footpad thickness, with a significantly reduction one week after the end of treatment, at which point no significant difference was found in footpads treated with Sb and SSPHE.

**Fig. 3:**
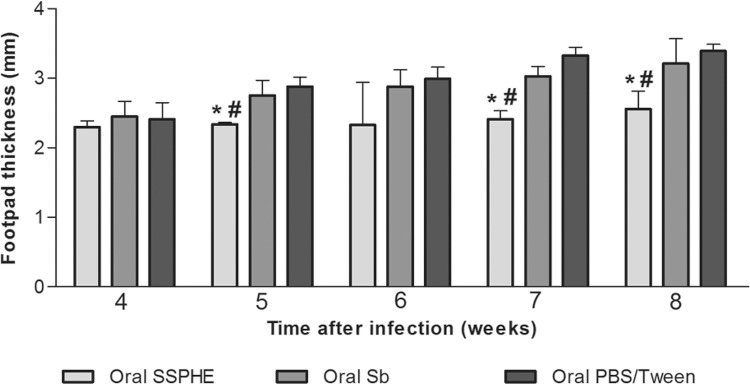
: kinetics of cutaneous lesion induced by *Leishmania amazonensis* after treatment with polar hydroethanolic extract from *Selaginella sellowii* (SSPHE) administered by the oral route (50 mg/kg/day during 20 days). Controls received*N*-methylglucamine antimonate (Sb) or phosphate-buffered saline (PBS)/Tween by the same route. Hamsters were infected in the left hind footpad with *L. amazonensis*promastigotes and treatment started four weeks after infection, ending seven weeks after infection. The data represent the mean ± standard deviation of 15 animals per group. *: p < 0.05 for SSPHE-treated vs. control animals (PBS/Tween); #: p < 0.05 for SSPHE-treated vs. Sb-treated group. Student’s *t* test.

Treatment with SSPHE administered orally resulted in a significant lesser footpad thickness in comparison to that of untreated animals, especially one week after the end of treatment (Fig. 3). The group that received Sb through the same administration route exhibited a progressive increase in footpad thickness. Moreover, no reduction in footpad thickness was found in the Sb-treated and untreated groups one week after the end of treatment (Fig. 3). No significant difference in footpad thickness was found between the animals that received Sb by the oral route and untreated animals.

*Effect of SSPHE treatment on parasite load* - Treatment with SSPHE by the intralesional route led to a significant reduction in parasite burden at the infection site in comparison to the untreated group. Indeed, no promastigotes were found in the serial dilution of the organs analysed, indicating an SI of 100% (Table II). The same result was observed in animals treated with Sb by the intralesional route. Oral treatment with SSPHE and Sb also induced a significant reduction in parasite burden at the infection site in comparison to the group that received PBS/Tween (99.2 and 98.5%, respectively). Both treatments through both administration routes induced a reduction in the weight of the infected footpads in comparison to the untreated group, especially in animals treated with SSPHE through the intralesional route (Table II).

**TABLE II:** Effect of polar hydroethanolic extract from *Selaginella sellowii* (SSPHE) (50 mg/kg) administered by oral and intralesional routes in hamsters infected with *Leishmania amazonensis* one week after the end of treatment

Drug (dosage)	Route of administration	Organ weight (g) (mean ± SD)	Supression of organ weight (%)	Suppression of parasite burden in the organ (%)	Mean number of parasites in organ/ng
Footpad					
None (control PBS/Tween)	Intralesional	0.43 ± 0.04	-	-	6.6
SSPHE (50 mg/Kg for 5 days)	Intralesional	0.32 ± 0.19	- 26	- 100	0
Sb (28 mg/kg)	Intralesional	0.38 ± 0.02	- 12	- 100	0
None (control PBS/Tween)	Oral	0.39 ± 0.03	-	-	4.9 x 10^2^
SSPHE (50 mg/Kg for 5 days)	Oral	0.36 ± 0.02^*a*^	- 7	- 99.2	3.9
Sb (28 mg/kg)	Oral	0.61 ± 0.23	- 2	- 98.5	9.7
Lymph node					
None (control PBS/Tween)	Intralesional	0.0 5± 0.009	-	-	1.6
SSPHE (50 mg/Kg for 5 days)	Intralesional	0.01^*a,b*^	+ 78	100	0
Sb (28 mg/kg)	Intralesional	0.03 ± 0.02	- 40	- 100	0
None (Control PBS/Tween)	Oral	0.03 ± 0.02	-	-	2.3
SSPHE (50 mg/Kg for 5 days)	Oral	0.08 ± 0.03^*a,b*^	+ 166	- 98.9	0.026
Sb (28 mg/kg)	Oral	0.05 ± 0.02	- 33	- 89.5	0.24

*a*: p < 0.05 for treated vs. positive control [phosphate-buffered saline (PBS)/Tween]; *b*: p < 0.05 for treated when compared to *N-*methylglucamine antimonate (Sb) group (ANOVA/Tukey); SD: standard deviation. Values represent the mean ± SD (n = 4).

In the popliteal draining lymph nodes, complete suppression of the parasite load occurred one week after treatment with SSPHE and Sb through the intralesional route. However, treatment with SSPHE induced an increase in the weight of these organs, while Sb treatment induced a 40% reduction in weight. Through the oral route, SSPHE also induced an increase in the weight, with a 98.9% reduction in the parasite load, whereas Sb treatment led to a reduction in lymph node weight, with an 89.5% reduction in the parasite load (Table II).

**Fig. 4:**
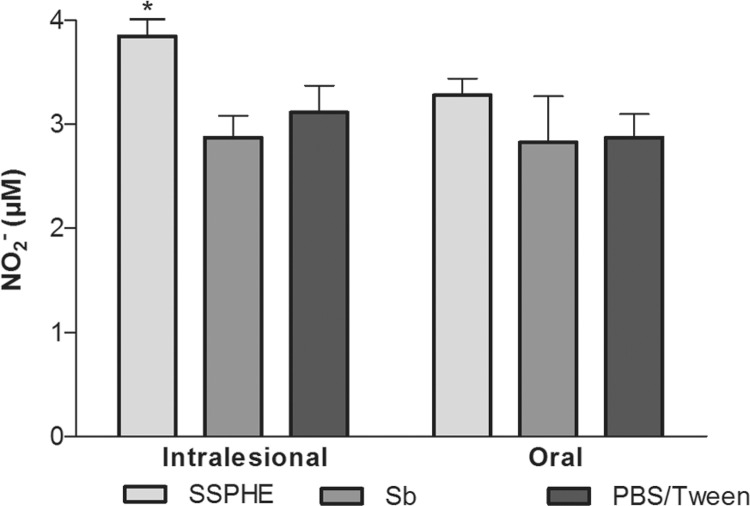
: effect of oral and intralesional treatment with polar hydroethanolic extract from Selaginella sellowii (SSPHE) (50 mg/kg) on nitric oxide (NO) production by peritoneal cells isolated from Leishmania amazonensis-infected hamsters. N-methylglucamine antimonate (Sb) and phosphate-buffered saline (PBS)/Tween were used as controls. The data represent the mean and ± standard deviation of four animals per group. Asterisk means p < 0.05 for SSPHE intralesional treatment vs. Sb intralesional treatment. Student’s t test.

*Effect of SSPHE on NO production* - Treatment with SSPHE through the intralesional route induced a significant increase in NO production by peritoneal cells derived from infected animals in comparison to the group treated with Sb. Treatment with SSPHE through the oral route also induced an increase in NO production in comparison to the groups that received Sb and PBS/Tween, but this increase did not achieve statistical significance (Fig. 4).

**Fig. 5:**
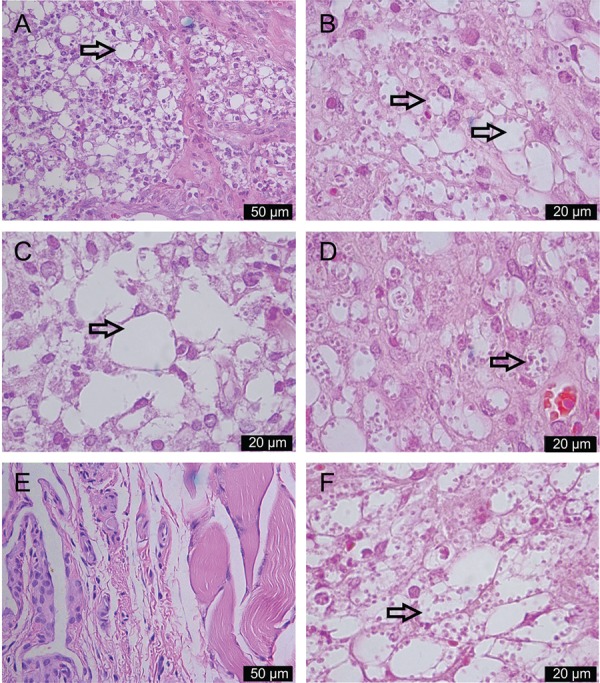
: histopathological study of the site of infection in hamsters infected in the left hind footpad with *Leishmania amazonensis* promastigotes and treated with polar hydroethanolic extract from *Selaginella sellowii*(SSPHE) (50 mg/kg) by intralesional and oral routes (A, C). Vacuolated macrophages with rare amastigotes are observed (arrows). The tissue fragments were obtained seven days after the end of treatments. Control nontreated group received phosphate-buffered saline/Tween by the same routes (B, D). There is a mononuclear infiltrate in the dermis, composed mainly of parasitised, vacuolated macrophages (arrows). Animals treated with *N-*methylglucamine by intralesional route showed nonparasitised tissue (E); by the oral route, several heavily infected macrophages (arrow) are observed (F). The figures are representative of five animals analysed in each group. Haematoxylin-eosin staining (A-F) 400X (A, E) and 1,000X (B-D, F) magnification.

*Histopathological study* - Footpads treated with SSPHE through the intralesional route revealed few cells with amastigotes. In contrast, numerous parasitised macrophages were observed in the control group (Fig. 5A, B). Infection associated with intralesional treatment resulted in intense inflammatory infiltrate composed of mononuclear cells and a few granulocytes. A few parasites were found in the footpads of animals that received SSPHE through the oral route (Fig. 5C). The inflammatory infiltrate in this case was composed of mononuclear cells. Animals treated with Sb by intralesional route showed nonparasitised tissue (Fig. 5E); by the oral route, however, several heavily infected macrophages were observed (Fig. 5F).

## DISCUSSION

Biflavonoids are a frequent class in the genus *Selaginella* and have been considered a chemical marker for this genus (Silva et al. 1995, Aguilar et al. 2013, Schulz et al. 2013). Recently, Rizk et al. (2014) have isolated the biflavonoids amentoflavone and robustaflavone from *S. selowii*. In the present work, four biflavonoids were identified in SSPHE. Amentoflavone (31.8 min) and robustaflavone (32.4 min) are C-link flavone dimmers and the other minor biflavonoids are O-linked flavones (34.3 min hinokiflavone and 35.9 min OMe-hinokiflavone). Romani et al. (2002) have demonstrated that O-linked and methylated biflavonoids are more likely to be retained in a C-18 column than C-linked ones, what is in agreement with the observed retention times.

Other compounds detected in the extract were the caffeoyl-hexoside derivatives. The lower retention time of this class suggests the presence of polar groups in the molecules. Correlation of retention time with physicochemical properties was demonstrated in several models (Tellez et al. 2009, Eugster et al. 2014). Caffeic acid linked to sugar groups has been described in the literature (Hamerski et al. 2005) however the number of sugars is limited to three units. In the present work, compounds with four, five, or six units of hexose linked to four, five, or six units of caffeic acid were found in SSPHE. Partial structure determination was based on the fragments obtained from high resolution MS/MS spectrum; all compounds exhibited sequential losses of the hexose/caffeic acid moiety. Above described data together with the molecular formula allowed the putative identification of these compounds as caffeoyl-hexoside derivatives. This is the first relate of these compounds in the literature.

The in vitro antileishmanial activity of SSPHE on intracellular amastigotes was satisfactory and proved not to be cytotoxic to the mammalian cells tested (Rizk et al. 2014). Thus, the extract was used for in vivo testing. It is important to note that SSPHE administered orally at a very high dose (2 g/Kg) did not cause acute toxicity in the animals (unpublished observations).

The treatment schedule in the present study was similar to that described by Fournet et al. (1996) and Patrício et al. (2008). It is important to note that no parasitic forms were detected in the infection site or draining lymph nodes using the limiting dilution method in animals treated with SSPHE through the intralesional administration route, suggesting that the extract reduces the parasite load by 100%. The overall reduction in parasite load has been described by Arruda et al. (2009), who demonstrated the in vitro and in vivo antileishmanial activity of limonene, which is a cyclohexanoid monoterpene found in the oil of citric plants.

We demonstrated that the intralesional injection of SSPHE reduced the parasite load in the infected footpads and draining lymph nodes, but also induced a significant, progressive increase in footpad thickness throughout treatment, whereas oral treatment with SSPHE led to a significant reduction in both parasite load and footpad thickness. The progressive increase in the footpad lesions may have resulted from a pro-inflammatory effect induced by the intralesional injection of SSPHE, together with the inflammatory response to the infection itself. This phenomenon has been described by Patrício et al. (2008), who also found a reduction in parasite burden despite the increase in footpad thickness, after the intralesional administration of a crude hydroalcoholic extract from*Chenopodium ambrosioides* (rich in flavonoid and terpenoid compounds) in mice infected with *L. (L.) amazonensis*. In the present work, histopathological study corroborated this hypothesis, revealing that the intralesional treatment with SSPHE induced an intense inflammatory infiltrate composed mainly of mononuclear cells. It should be stressed, however, that footpads treated with an intralesional injection of SSPHE exhibited a significant reduction in thickness one week after the end of treatment in comparison to nontreated footpads, returning to values similar to those measured prior to infection. In this same timeframe, no significant differences were found between the Sb and SSPHE groups submitted to the intralesional route. However, no significant difference was found in footpads treated with orally administered Sb in comparison to untreated footpads, while a significant reduction in footpad thickness was found among those treated with orally administered SSPHE in comparison to controls.

A number of authors have shown that biflavonoids are responsible for the antileishmanial activity in plant extracts (Sharma et al. 2003, Weniger et al. 2006,Kunert et al. 2008). Rizk et al. (2014) observed a higher in vitro antileishmanial activity of the biflavonoids isolated (amentoflavone or robustaflavone) compared to the one of the whole extract. In the present work, however, the great in vivo activity may be due to a synergistic action of the compounds. Indeed, the presence of caffeoyl-hexoside derivatives associated to biflavonoids could immunostimulate the animals and booster the response. Zeng et al. (2008) demonstrated an immunopotentiation effect from a caffeoyl-glycoside. This compound stimulated in vitro proliferation of peritoneal macrophages and increased CD4^+^ and CD8^+^ populations. At the same time, increased interleukin (IL)-2, IL-12 and interferon-gamma cytokines were found, unlike decreased IL-4 and IL-10, evincing the T-helper 1 profile classically associated with protection in leishmaniasis. This class of compounds could be also active against*Leishmania*, as established by Abdel-Mageed et al. (2012), who found phenylpropanoid glycosides active against *Leishmania (Leishmania) donovani* promastigotes.

The presence of biflavonoids and caffeoyl-hexoside derivatives in SSPHE suggests an immunomodulatory action from these compounds associated with the control of infection. The increase in NO production by peritoneal cells isolated from animals treated with SSPHE by the intralesional route corroborates the immunomodulatory activity toward resistance to the parasite. The stimulation of NO production in murine macrophages infected with *L. (L.) amazonensis* has already been described elsewhere as an inhibitory effect of a treatment of plant origin (Pereira et al. 2005). The increased NO production induced by SSPHE in vivo corroborates the findings of Rizk et al. (2014), who showed the increase in NO production by peritoneal macrophages infected and treated by SSPHE in vitro.

Gupta et al. (1992) evaluated different experimental models for leishmaniasis and found that practically no treatment schedule provides adequate information for understanding the overall effectiveness of a potential antileishmanial drug, once it depends on the interaction between the parasite and the immune system. Indeed, it is well documented that the cure of animals from infection occurs due to the combined effect of drug action and immunological status (Sacks et al. 1987).

The present study demonstrated the in vivo activity of the hydroethanolic extract from *S. sellowii* when administered through the intralesional and oral routes. Besides compounds of specific antileishmanial activity, the extract holds compounds, which could enhance the immune response against the parasite. This is a desirable characteristic for a candidate drug for the treatment of cutaneous leishmaniasis. Further studies with purified fractions have been carried out to establish which compound is responsible for the immunomodulatory properties.
